# The Adherence to Initial Processes of Care in Elderly Patients with Acute Venous Thromboembolism

**DOI:** 10.1371/journal.pone.0100164

**Published:** 2014-07-01

**Authors:** Anna K. Stuck, Marie Méan, Andreas Limacher, Marc Righini, Kurt Jaeger, Hans-Jürg Beer, Joseph Osterwalder, Beat Frauchiger, Christian M. Matter, Nils Kucher, Michael Egloff, Markus Aschwanden, Marc Husmann, Anne Angelillo-Scherrer, Nicolas Rodondi, Drahomir Aujesky

**Affiliations:** 1 Division of General Internal Medicine, Bern University Hospital, Bern, Switzerland; 2 CTU Bern, Department of Clinical Research, and Institute of Social and Preventive Medicine, University of Bern, Bern, Switzerland; 3 Division of Angiology and Hemostasis, Geneva University Hospital, Geneva, Switzerland; 4 Division of Angiology, Basel University Hospital, Basel, Switzerland; 5 Cantonal Hospital of Baden, Baden, Switzerland; 6 Emergency Department, Cantonal Hospital of St. Gallen, St. Gallen, Switzerland; 7 Department of Internal Medicine, Cantonal Hospital of Frauenfeld, Frauenfeld, Switzerland; 8 Cardiovascular Research, Institute of Physiology, Zurich Center for Integrative Human Physiology, University of Zurich, Zurich, Switzerland; 9 Division of Angiology, Bern University Hospital, Bern, Switzerland; 10 Division of Diabetology, Geneva University Hospital, Geneva, Switzerland; 11 Clinic of Angiology, Zurich University Hospital, Zurich, Switzerland; 12 University Clinic of Hematology and Hematology Central Laboratory, Bern University Hospital, Bern, Switzerland; 13 Division of Cardiology, Zurich University Hospital, Zurich, Switzerland; Maastricht University Medical Center, Netherlands

## Abstract

**Background:**

We aimed to assess whether elderly patients with acute venous thromboembolism (VTE) receive recommended initial processes of care and to identify predictors of process adherence.

**Methods:**

We prospectively studied in- and outpatients aged ≥65 years with acute symptomatic VTE in a multicenter cohort study from nine Swiss university- and non-university hospitals between September 2009 and March 2011.

We systematically assessed whether initial processes of care, which are recommended by the 2008 American College of Chest Physicians guidelines, were performed in each patient. We used multivariable logistic models to identify patient factors independently associated with process adherence.

**Results:**

Our cohort comprised 950 patients (mean age 76 years). Of these, 86% (645/750) received parenteral anticoagulation for ≥5 days, 54% (405/750) had oral anticoagulation started on the first treatment day, and 37% (274/750) had an international normalized ratio (INR) ≥2 for ≥24 hours before parenteral anticoagulation was discontinued. Overall, 35% (53/153) of patients with cancer received low-molecular-weight heparin monotherapy and 72% (304/423) of patients with symptomatic deep vein thrombosis were prescribed compression stockings. In multivariate analyses, symptomatic pulmonary embolism, hospital-acquired VTE, and concomitant antiplatelet therapy were associated with a significantly lower anticoagulation-related process adherence.

**Conclusions:**

Adherence to several recommended processes of care was suboptimal in elderly patients with VTE. Quality of care interventions should particularly focus on processes with low adherence, such as the prescription of continued low-molecular-weight heparin therapy in patients with cancer and the achievement of an INR ≥2 for ≥24 hours before parenteral anticoagulants are stopped.

## Introduction

The incidence of acute venous thromboembolism (VTE), defined as acute deep vein thrombosis (DVT) or pulmonary embolism (PE), rises exponentially with age [1], [2]. In the geriatric population, VTE not only carries a higher mortality but also a higher rate of VTE recurrence and major bleeding than in younger patients [3].

The American College of Chest Physicians (ACCP) Evidence-based Clinical Practice Guidelines recommend specific processes of care for the management of patients with acute VTE [4], [5]. Several of these processes have the potential to improve patient outcomes and to reduce the length of hospital stay and health care costs [6]–[12]. These recommended processes include the administration of parenteral anticoagulation for at least five days, initiation of oral anticoagulation on the first treatment day, maintenance of an International Normalized Ratio (INR) ≥2 for at least 24 hours before parenteral anticoagulation is discontinued, continued therapy with low-molecular-weight heparin (LMWH) in patients with cancer, and the use of compression stockings in patients with symptomatic DVT [4], [5]. Prior studies demonstrated wide practice variation and suboptimal adherence to these processes of care [13]–[15].

Despite the higher VTE incidence and complication rates in elderly patients, to our knowledge, only two retrospective studies have examined the adherence to VTE-related processes of care in patients aged ≥65 years [16], [17]. In a large multicenter prospective cohort study, we therefore assessed whether elderly patients aged 65 years or over with VTE received recommended processes of care in the early phase of VTE and to identify predictors of process adherence.

## Methods

### Ethics statement

We asked eligible patients to provide written informed consent. The study was approved by the Institutional Review Board of each participating site (Commission cantonale (VD) d'éthique de la recherche sur l'être humain, Commission cantonale d'éthique de la recherche, Kantonale Ethikkommission Bern, Kantonale Ethikkommission Zürich, Kantonale Ethikkommission Kanton Aargau, Ethikkommission des Kanton St. Gallen, Ethikkommission des Kantons Thurgau, Ethikkommission Luzern, Ethikkommission Basel). The committees approved the consent procedure of participants.

### Cohort sample

The study was conducted between September 1, 2009 and March 31, 2011 as part of a prospective, multicenter cohort study to assess medical outcomes of patients aged ≥65 years with acute, symptomatic VTE from all five Swiss university and four high-volume non-university hospitals [18]. Potential participants were consecutively identified in the inpatient and outpatient services of all participating study sites. We defined DVT as the acute onset of leg pain or swelling plus incomplete compressibility of a venous segment on ultrasonography or an intraluminal filling defect on contrast venography) [19]. Because the iliac vein and the inferior vena cava may be technically difficult to compress, iliac/caval DVT was defined as abnormal duplex flown patterns compatible with thrombosis or an intraluminal filling defect on contrast computed tomography or magnetic resonance imaging venography [20]. Given that ultrasonography has a reduced sensitivity and specificity for distal DVT [21] patients with distal DVT were included only if the incompressible distal vein transverse diameter was at least 5 mm. We defined PE as the acute onset of dyspnea, chest pain, or syncope coupled with a new high-probability ventilation/perfusion lung scan; a new contrast filling defect on spiral computed tomography or pulmonary angiography; or the new documentation of a proximal DVT either by venous ultrasound or contrast venography [22], [23]. Radiographic studies used to diagnose VTE were interpreted by on-site vascular specialists or radiologists.

Exclusion criteria were inability to provide informed consent (i.e., severe dementia), conditions incompatible with follow-up (i.e., terminal illness or place of living too far away from the study center), insufficient German or French speaking ability, thrombosis at a different site than lower limb, catheter-related thrombosis, or previous enrollment in the cohort. We also excluded patients who received thrombolytic therapy, surgical treatment (i.e., thrombo-/embolectomy), or a vena cava filter.

Treatment of VTE, e.g., the type of anticoagulant used (i.e., parenteral anticoagulant followed by vitamin K antagonists or parenteral anticoagulation alone) and the prescription of compression stockings, was entirely left to the discretion of the managing physicians.

### Baseline data collection

For all enrolled patients, baseline demographic information (age, gender, weight, height, level of education and place of living), type (PE, DVT, or both) and occurrence of VTE (hospital-acquired vs. home-acquired), comorbid conditions (history of major bleeding, cardiovascular comorbidity, diabetes mellitus, renal impairment), laboratory findings (anemia, low platelet count), concomitant use of platelet inhibitors, and the number of concomitant drug treatments were prospectively collected by medical record review by trained research nurses and recorded on standard data collection forms. Information was also gathered on pharmacological (type, timing, and duration of anticoagulant treatment) and non-pharmacological treatments (prescription of compression stockings).

### Processes of care

We assessed whether the following five processes of care, which were recommended in the 2008 ACCP guidelines [4], were performed in each patient: (1) administration of parenteral anticoagulants for ≥5 days; (2) start of oral anticoagulation on the first treatment day (defined as the administration of the first dose of vitamin K antagonists within 24 hours after VTE diagnosis); (3) continuation of parenteral anticoagulants until an international normalized ratio [INR] ≥2 for at least 24 hours; (4) continued use of LMWH without switching to vitamin K antagonists in patients with an active cancer (defined as active solid or hematologic cancers requiring chemotherapy, radiotherapy, or surgery within the previous three months); and (5) prescription of compression stockings in patients with symptomatic DVT. For our analysis of processes of care 1–3, we considered only patients who were switched from parental anticoagulants to vitamin K antagonists within 21 days. We collected data up to 90 days following the index VTE.

### Statistical analyses

Baseline characteristics are shown as numbers and percentages. Analyzed and non-analyzed patients were compared using the chi-squared test or the Wilcoxon rank-sum test as appropriate. For each of the five processes of care, as well as for the combination of the three anticoagulation-related processes of care, the number and proportion of guideline adherence was calculated.

We used logistic regression to explore associations between baseline patient characteristics shown in Table 1 and the adherence to three processes of care: (1) duration of parenteral anticoagulation ≥5 days, (2) start of oral anticoagulation on the first treatment day, and (3) continuation of parenteral anticoagulation until the INR is ≥2 for 24 hours. The sample sizes of the subpopulations with cancer and symptomatic DVT were not large enough to conduct robust multivariable analyses. For missing values, we performed multiple imputation by chained equations [24], assuming missing data to be missing at random. A total of twenty imputed datasets were generated based on all baseline variables, the three outcome variables and study site. Adjusted odds ratios (OR) and corresponding 95% confidence intervals (CI) and P-values were calculated applying Rubin's rules [25]. Adjustment was done for all baseline variables as fixed effects and study site as a random effect in a mixed-effects logistic model. In a sensitivity analysis, a complete case analysis was performed, excluding any cases with missing values. All analyses were performed using STATA statistical software, Version 12.0.

**Table 1:** Patient Baseline Characteristics

Characteristic	n (%)a)
Age>80 years	261 (28)
Female sex	443 (47)
BMI>30 kg/m^2^	226 (24)
Nursing home care	24 (3)
Higher level of educationalb)	427 (45)
Symptomatic PEc)	654 (69)
Hospital-acquired VTEd)	175 (18)
History of major bleeding	92 (10)
Active cancere)	153 (16)
Cardiovascular comorbidityf)	231 (24)
Diabetes mellitus	147 (15)
Renal impairmentg)	197 (21)
Anemiah)	374 (39)
Low platelet count (<150×10^9^/L)	131 (14)
Concomitant antiplatelet medicationi)	315 (33)
Polypharmacyk)	489 (51)

Abbreviations: BMI = body mass index; PE = pulmonary embolism; VTE = venous thromboembolism.

a) Overall, missing values were 0.5% for BMI, 0.1% for nursing home care, 0.2% for higher level of education, 0.1% for history of major bleeding, 6.6% for hemoglobin level, and 6.6% for platelet count.

b) High school or post high school attendance.

c) Symptomatic PE with or without a deep vein thrombosis.

d) VTE event occurring during a hospital stay.

e) Requiring chemotherapy, radiotherapy, surgery, or palliative care during the last three months.

f) Acute heart failure or history of heart failure or coronary heart disease.

g) Glomerular filtration rate <30 ml/min or a history of chronic renal failure.

h) Hemoglobin level <13 g/dL for men or <12 g/dL for women.

i) Use of aspirin, clopidogrel, prasugrel, and/or dipyridamol.

k) Concomitant use of more than four drugs.

(n = 950).

## Results

### Patient identification and baseline characteristics

**Figure 1:**
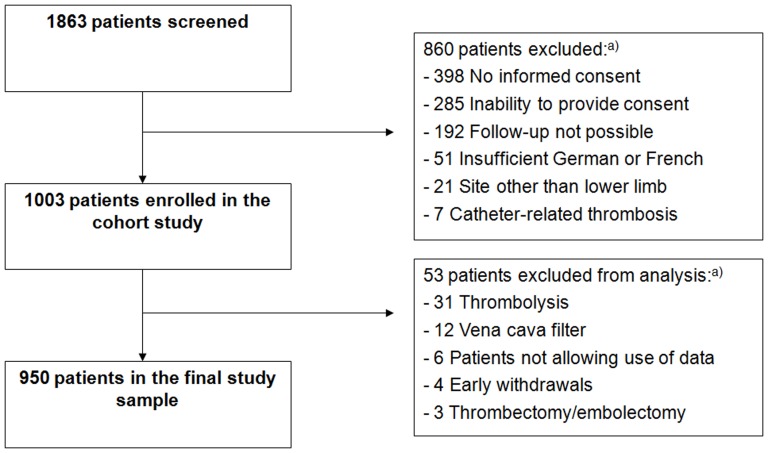
Flow chart. Abbreviations: DVT = deep vein thrombosis. A) Multiple reasons for exclusion may apply.

Of 1863 patients screened during the study period, 1003 were originally enrolled in the cohort study (Figure 1). Of these, 53 were excluded, leaving a study sample of 950 analyzed patients. Non-analyzed patients were statistically significantly older (mean age 77 vs. 76 years; P<0.001) and more likely to be women (59% vs. 47%; P<0.001) than analyzed patients.

Overall, 28% of patients were aged >80 years, 47% (443/950) were women, and 3% (24/950) were nursing home residents (Table 1). Eighteen percent (175/950) had hospital-acquired VTE. Of the 775 patients who developed VTE in an outpatient setting (community-acquired VTE), 589 (76%) were admitted to the hospital.

### Adherence to recommended processes of care

Overall, 750 patients were switched from parental anticoagulants to vitamin K antagonists. Of these, 86% (645/750) received parenteral anticoagulation for ≥5 days, 54% (405/750) had oral anticoagulation started on the first treatment day, and 37% (274/750) had an INR ≥2 for ≥24 hours before parenteral anticoagulation was discontinued (Table 2). Only 18% (138/750) of patients received all three anticoagulation-related processes of care.

**Table 2:** Adherence to Recommended Processes of Care.

Processes of care	n/N (%)a)
1) Duration of parenteral anticoagulation ≥5 days	645/750 (86)
2) Start of oral anticoagulation on the first treatment day	405/750 (54)
3) Parenteral anticoagulation until an INR ≥2 for at least 24 hours	274/750 (37)
4) Continued treatment with LMWH for active cancer	53/153 (35)
5) Prescription of compression stockings for symptomatic DVT	304/423 (72)

Abbreviations: INR = international normalized ratio; LMWH = low-molecular-weight heparin; DVT = deep vein thrombosis.

a) Denominators change because of different subgroup analyses.

For instance, for processes of care 1–3, we analyzed the 750 patients who were switched from LMWH to oral anticoagulants. For processes of care 4 and 5, we analyzed the 153 with cancer and the 423 patients with symptomatic DVT, respectively.

Among the 153 patients with active cancer, only 35% (53/153) received continued LMWH, 25% (39/153) received unfractionated heparin or fondaparinux followed by oral anticoagulation, 23% (35/153) unfractionated heparin or fondaparinux alone, 14% (21/153) LMWH followed by oral anticoagulation, 2% (3/153) oral anticoagulation alone, and 1% (2/153) no anticoagulation at all. Of 423 patients who had symptomatic DVT, 72% (304/423) were prescribed compression stockings.

There was some variation by type of VTE. As shown in Table 3, patients with PE were more likely to have an INR ≥2 for ≥24 hours before parenteral anticoagulation was stopped than patients with DVT only (40% vs. 27%; P<0.001). On the other hand, patients with cancer who had DVT only were more likely to receive continued treatment with LMWH than patients with PE (52% vs. 26%; P = 0.001).

**Table 3:** Stratified Analyses by Type of Venous Thromboembolism.

	Overt PEa) n/N (%)	DVT only n/N (%)	P-value
Parenteneral anticoagulation for ≥5 days	465/546 (85)	180/204 (88)	0.281
Initiation of oral anticoagulation on the first treatment day	283/546(52)	122/204 (60)	0.051
INR ≥2 during at least 24 hours before discontinuation of parenteral anticoagulation	219/546 (40)	55/204 (27)	<0.001
Continued treatment with LMWH for cancer	26/101 (26)	27/52 (52)	0.001
Prescription of compression stockings for symptomatic DVT	88/127 (69)	216/296 (73)	0.440

Abbreviations: PE = pulmonary embolism; DVT = deep vein thrombosis; INR = international normalized ratio; LMWH = low-molecular-weight heparin.

a) With or without concomitant DVT.

There was a substantial variation across study sites: adherence varied from 79% to 94% for parenteral anticoagulation for ≥5 days, from 47% to 62% for the start of oral anticoagulation on the first treatment day, from 13% to 50% for an INR ≥2 for ≥24 hours before parenteral anticoagulation was discontinued, from 0% to 64% for continued LMWH in patients with cancer, and from 61% to 90% for the prescription of compression stockings in patients with symptomatic DVT.

### Predictors of process adherence

**Figure 2:**
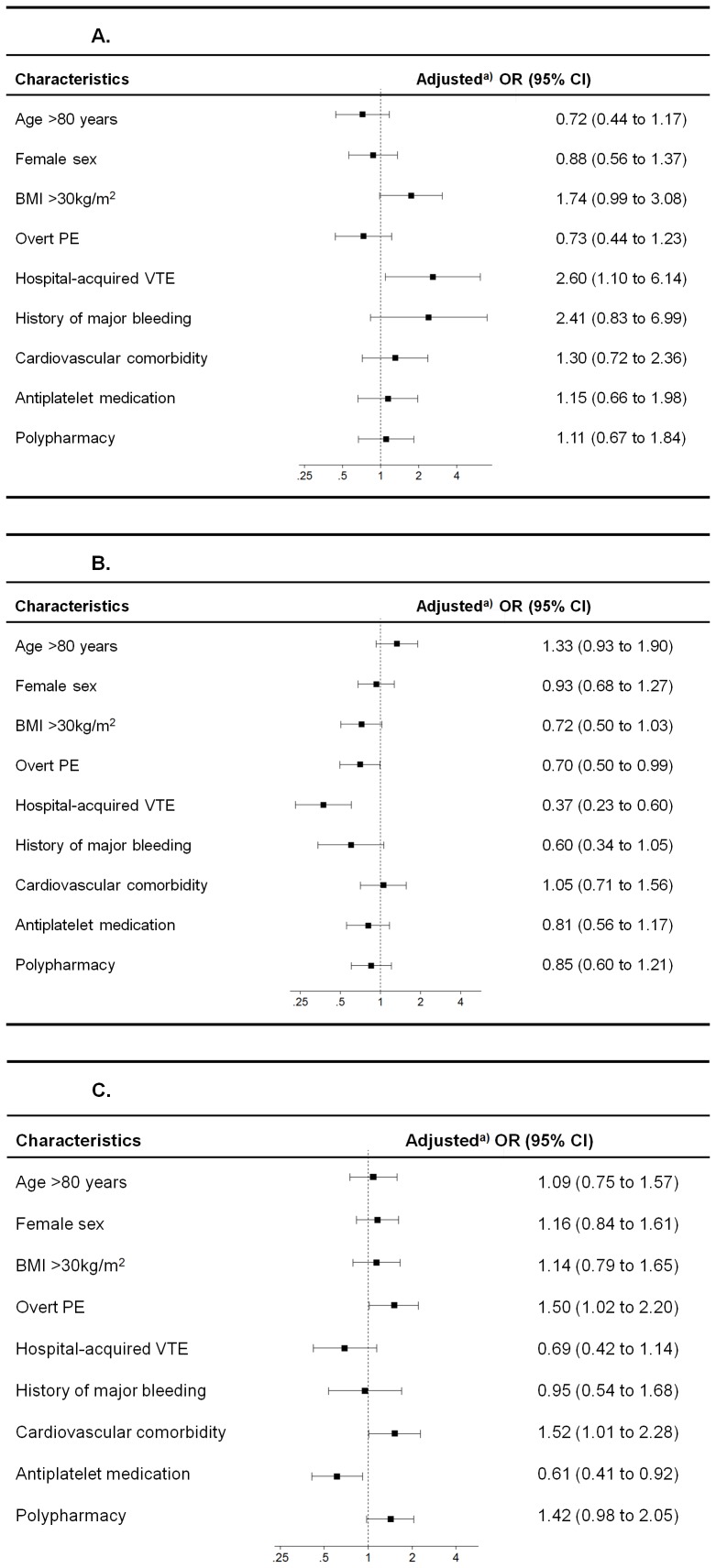
A. Predictors for using parenteral anticoagulation for ≥5 days (n = 750). Abbreviations: OR = odds ratio; CI = confidence interval; BMI = body mass index; VTE = venous thromboembolism; PE = pulmonary embolism. A. a) Adjusted for all baseline characteristics shown in Table 1 as a fixed effect and for study site as a random effect. A selection of variables is displayed in the figures to improve legibility i.e. age, sex and all variables with statistical significant numbers. **B. Predictors for starting oral anticoagulation on the first treatment day (n = 750). C. Predictors for continuing parenteral anticoagulation until an international normalized ratio of ≥2 for 24 hours (n = 750).**

In multivariable analyses, patients with hospital-acquired VTE (OR, 2.60; 95% CI, 1.10–6.14) were statistically significantly more likely to receive parenteral anticoagulation for ≥5 days (Figure 2A). Patients with hospital-acquired VTE (OR, 0.37; 95% CI, 0.23–0.60) and overt PE (OR, 0.70; 95% CI, 0.50–0.99) were less likely to receive oral anticoagulation on the first treatment day (Figure 2B). Finally, patients with overt PE (OR, 1.50; 95% CI, 1.02–2.20) and cardiovascular comorbidity (OR, 1.52; 95% CI, 1.01–2.28) were more likely to continue parenteral anticoagulation until the INR was ≥2 for 24 hours, whereas patients with concomitant antiplatelet medication were less likely to have two INR values ≥2 for 24 hours before parenteral anticoagulation was stopped (OR, 0.61; 95% CI, 0.41–0.92) (Figure 2C). Age >80 years was not a significant predictor of process adherence. When patients with missing values were excluded from analyses, results remained similar.

## Discussion

In this prospective multicenter cohort study of 950 elderly patients with acute symptomatic VTE, we found that adherence to five recommended processes of care was variable and partially suboptimal. The adherence rate varied from 86% for treatment with parenteral anticoagulants for at least five days to a mere 35% for continued treatment with LMWH in patients with active cancer. Overall, our findings are consistent with prior studies demonstrating that the use of recommended processes of care and the achievement of treatment goals for patients with VTE leaves room for improvement [6], [13]–[17], [26].

If anything, adherence rates were even lower in our elderly population (mean age 76 years) for several processes of care than in previous studies enrolling younger patients with VTE (mean age 56–66 years) [6], [13]–[15]. Compared to a retrospective study of U.S. veterans in which oral anticoagulation was initiated on the first treatment day in 64% of cases [6], we found a substantially lower rate in our study sample (54%). In a prospective study of Canadian outpatients with VTE [15], 60% of patients with cancer-related VTE received LMWH monotherapy compared to 35% in our study. While the proportion of patients who had parenteral anticoagulation continued until an INR value ≥2.0 for 24 hours was 37% only in our sample, this target was achieved in about 50% of patients in younger patients [13], [14].

Our results demonstrating a low adherence rate to anticoagulation-related processes of care in elderly patients are consistent with prior evidence showing that the rate of anticoagulation is low in hospitalized elderly patients with atrial fibrillation [27]. Elderly, multimorbid medical inpatients have also a low rate of venous thromboprophylaxis [28]. It remains unclear whether the low adherence rate is due to lack of physicians' knowledge, physicians' fear to induce bleeding, the presence of comorbid conditions that are not compatible with guideline adherence, or patient preferences [29]. For instance, elderly patients with cancer who develop VTE are more likely to have severe renal failure and therefore, may not be candidates for extended LMWH treatment. Similarly, although LMWHs seem to be acceptable to patients with cancer [30], many elderly patients may prefer to receive oral anticoagulants rather than daily injections with LMWH. One study found that 30% of physicians did not believe it is necessary to have a therapeutic INR for ≥2 days before discontinuing heparin [14].

Prior evidence about the effect of age on VTE-related anticoagulation practices is limited. While age did not influence the duration of anticoagulant therapy in one study [16], older patients were more likely to have continuation of parenteral anticoagulation until INR was ≥2 for 24 hours in another study [6]. In an Italian registry, compression stockings were 1.6 times more likely to be prescribed in patients aged 40–60 years than in patients aged >80 years [31].

To our knowledge, there are no studies examining the effectiveness of interventions to improve anticoagulation-related processes of care for established VTE. Although evidence from studies on VTE prophylaxis suggests that active, multifaceted strategies, including provider education, electronic alerts, and regular audit and feedback to medical staff, appear to be the most promising interventions [29], [32]–[34], whether such interventions have the potential to improve therapeutic anticoagulation-related care and outcomes of VTE must be further examined.

We could not identify a clear pattern of consistent predictors of suboptimal process adherence that may become potential targets for quality improvement interventions. While hospital-acquired VTE was associated with delayed initiation of oral anticoagulation, patients with the same condition were more likely to get parenteral anticoagulation for ≥5 days. Given that hospitalized patients often undergo diagnostic or therapeutic procedures, parenteral anticoagulation is easier to control and thus possibly preferred. Similarly, patients with PE, who are hospitalized in most instances, were less likely to get oral anticoagulants on the first treatment day but were more likely to have two INR values ≥2.0 before parental anticoagulation was stopped. Our finding that patients with concomitant antiplatelet therapy were less likely to have two INR values before stopping parenteral anticoagulation may reflect physicians' underlying fear of bleeding in such patients [29].

Our study has potential limitations. First, our sample may not reflect the full spectrum of elderly patients with VTE because analyzed patients were younger and more likely to be men than non-analyzed patients. Moreover, given that patients were enrolled from hospital inpatient and outpatient services, patients with DVT who were diagnosed and entirely managed in the primary care or nursing home setting may be underrepresented in our sample. This could explain the relatively high proportion of patients who had symptomatic PE (69%) in our cohort. Because we excluded patients with severe dementia who may have a lower process adherence, our study may overestimate the true process adherence rates [35]. However, given that our multicenter cohort study enrolled a broad population of in- and outpatients aged ≥65 years from university- and non-university hospitals, our study sample should be fairly representative for elderly patients with VTE. Second, as treating physicians were aware that patients were included in a cohort study, they may have altered their behavior and better followed recommended processes of care. Thus, we cannot exclude that the adherence rates in our study may overestimate the true process adherence rates. Third, because data on physician characteristics (e.g., type and duration of training) were not available in our database, we could not examine whether these factors were associated with adherence to VTE-related processes of care. Similarly, we could not explore potential physician barriers to antithrombotic guideline adherence in elderly patients with VTE, which must be done in a further study. Fourth, because patient enrollment in our study started in 2009, we used the 2008 version of the ACCP guidelines as a benchmark for VTE-related quality of care and not the latest 2012 version [4], [5]. However, the recommendations with respect to the processes of care examined in our study are basically the same in both versions. Finally, our goal was to examine adherence rates to initial processes of care in elderly patients with VTE and to identify predictors of process adherence. Thus, we did not examine the impact of process adherence on patient outcomes, which must be done in another study.

In conclusion, adherence to recommended processes of care was variable and partially suboptimal in elderly patients with VTE. Additional efforts are needed to increase process adherence and quality of care in such patients. Quality of care interventions should particularly focus on recommended processes with low adherence rates, such as the prescription of continued LMWH in patients in cancer and the achievement of an INR ≥2 for ≥24 hours before parenteral anticoagulants are stopped.
